# Evaluation of the effect of the mouth gag use on optic nerve sheath diameter of pediatric patients undergoing tonsillectomy or Adenotonsillectomy: An observational study

**DOI:** 10.1186/s12871-020-01079-7

**Published:** 2020-07-03

**Authors:** Başak Altiparmak, Melike Korkmaz Toker, Ali İhsan Uysal, Sabri Köseoğlu, Semra Gümüş Demirbilek

**Affiliations:** 1 Department of Anesthesiology and Reanimation, Muğla Sıtkı Koçman University, Muğla, Turkey; 2 Department of Anesthesiology and Reanimation, Muğla Sıtkı Koçman University Training and Research Hospital, Muğla, Turkey; 3 Department of Ear Nose Throat, Muğla Sıtkı Koçman University, Muğla, Turkey

**Keywords:** Optic nerve, Tonsillectomy, Ultrasonography, Mouth gag

## Abstract

**Background:**

A mouth gag is usually used during tonsillectomy and adenotonsillectomy surgeries, cleft palate repair, obstructive sleep apnea surgery, and intraoral tumor excision. The placement of the gag causes hemodynamic changes similar to laryngoscopy. The aim of this study was to evaluate the effect of mouth gag placement on the optic nerve sheath diameter (ONSD) of pediatric patients. The secondary aim was to assess the relationship between neck extension and changes in ONSD.

**Methods:**

The trial was prospectively registered to the Australian New Zealand Clinical Trials Registry (Trial ID: ACTRN12618000551291) on 12.04.2018. This prospective, observational study was performed in a tertiary university hospital operating room between 01.05.2018–01.07.2018. Thirty-five children aged < 18 years, with ASA I status, who were scheduled for tonsillectomy and adenotonsillectomy surgeries were prospectively included in the study. Measurements of ONSD were performed (T0) after induction of anesthesia, (T1) after endotracheal intubation, (T2) after mouth gag placement, and (T3) 20 min after mouth gag placement. After the mouth gag was placed and the head was positioned for surgery, the degree of neck extension was calculated.

**Results:**

All participants completed the study. There were significant differences in ONSD values at time points T1, T2, and T3 (*p* < 0.001, CI: − 0.09,-0.05; *p* < 0.001, CI: − 0.09,-0.05; *p* < 0.001, CI: − 0.05,-0.02; respectively). The maximum increase in ONSD was after intubation (0.69 ± 0.06 mm) and immediately after mouth gag placement (0.67 ± 0.07 mm). ONSD values continued to increase 20 min after gag placement (0.36 ± 0.04). There was no relation between the degree of neck extension and ONSD values (β = 0.63, *p* = 0.715).

**Conclusions:**

The use of a mouth gag causes significant increases in ONSD measurements of children. Therefore, attention to the duration of mouth gag placement should be considered during surgery.

**Trial registration:**

The trial was prospectively registered to the Australian New Zealand Clinical Trials Registry (Trial ID: ACTRN12618000551291) on 12.04.2018.

## Background

The introduction of oral antibiotics in the 1960s dramatically decreased the rate of tonsillectomy (T) and adenotonsillectomy (AT) surgeries; however, T and AT remain as some of the most common surgeries performed in children worldwide. Traditionally, the head of the patient is positioned in extension and a mouth gag is placed for these surgeries [1]. The Crowe-Davis mouth gag was initially designed for mouth opening and intraoperative anesthetic agent delivery, then Boyle modified the original device to use the mouth gag with endotracheal tubes. The mouth gag has three parts: the blade has a central groove for the positioning of the endotracheal tube, a gag helps mouth opening, and lastly the suspension system of the gag maintains the position [2]. Although it provides advantage for access to the intraoral cavity, placement of a mouth gag results in hemodynamic changes similar to laryngoscopy, which cause significant increases in intracranial pressure (ICP) and intraorbital pressure (IOP) [3]. Moreover, excessive mouth opening causes tonic contractions in muscles of mastication and postoperative pain in the temporomandibular joint [4].

Several previous studies evaluated the ultrasonographic measurement of optic nerve sheath diameter (ONSD) as a non-invasive, simple and rapid way to detect pressure changes of the intracranial compartment [5, 6]. The sheath around the optic nerve is an anatomic extension of the dura mater, and within the sheath, the intracranial subarachnoid space extends through the optic nerve. Therefore, a rise in ICP is directly transmitted to the distensible subarachnoid space around the optic nerve. The transbulbar sonography technique for the estimation of ICP, which was first described by Helmke et al., is performed by measuring the ONSD of children [7]. To date, several studies have evaluated the reliability of ONSD measurements through concurrent magnetic resonance imaging (MRI) and invasive methods [8–10]. Steinborn et al. [11] observed 99 healthy children and adolescents in order to determine the normal values of ONSD. They reported that the mean value for ultrasonographic ONSD measurements was 5.75 ± 0.52 mm. One year later, the authors observed 56 children with normal ICP and 25 children with elevated ICP to determine a cut-off value for normal ONSD [8]. In this study, the diagnosis of elevated ICP (ICP ≥ 15 mmHg) was based on different invasive measurement methods such as intracranial devices or lumbar puncture, concurrent imaging studies, and ophthalmologic findings. The researchers reported that mean ONSD in patients with normal ICP was 5.77 ± 0.48 mm, and it was 6.85 ± 0.81 mm in children with elevated ICP. According to receiver operating characteristic (ROC) curve analysis, they calculated the optimal cut-off value of ONSD for predicting elevated ICP as 6.0 mm, with a sensitivity of 82% and specificity of 74%.

Although the measurement of ONSD has been used in different clinical scenarios in the current literature, no study has evaluated the effect of mouth gag placement on ONSD measurements. Accordingly, the primary aim of this study was to evaluate the effect of mouth gag placement on the ONSD of pediatric patients. The secondary aim was to assess the relationship between neck extension and changes in ONSD.

## Methods

This observational study was approved by Muğla Sıtkı Koçman University Clinical Research Ethic Committee (approval number: XII, 26.04.2018) and registered at anzctr.org.au (Trial ID: ACTRN12618000551291), and conducted in accordance with the current Declaration of Helsinki. Written informed consents were obtained from the parents of all children and verbal informed consents were obtained from the children themselves, who were aged over 6 years. Patients aged 3–18 years with American Society of Anesthesiologists (ASA) physical status I-II who were scheduled for a T or AT surgery were prospectively included in the study. The exclusion criteria were patients with known acute or chronic ophthalmic diseases, history of previous ophthalmic surgery, increased ICP, receiving ß blocker, calcium channel blocker, statin or nitrate treatment, more than one attempt for endotracheal intubation, and duration of mouth gag use < 20 min.

All children received preoperative medication with midazolam 0.5 mg kg^− 1^ orally (maximum dose of 15 mg) approximately 15–20 min prior to the induction of anesthesia. Standard monitoring was employed to all children with electrocardiography, non-invasive arterial blood pressure, pulse oximetry, bi-spectral index (BIS) (Datex-Ohmeda S/5 monitor M-BIS module, Helsinki, Finland), nasopharyngeal temperature, end-tidal CO_2_ (EtCO_2_) measurements, and gas analysis. Anaesthesia was induced using intravenous propofol 1–2 mg/kg, fentanyl 1 mcg/kg and rocuronium bromide 0.6 mg/kg. When the BIS score decreased to under 60, the patients were intubated by an experienced anaesthesiologist on the first attempt. Anesthesia was maintained with sevoflurane in 40% O2 and 60% air mixture, and the inspired concentration of sevoflurane was targeted to maintain a BIS score between 40 and 60. Peak inspiratory pressure was strictly maintained between 11 and 13 cm H_2_O so as not to affect ICP. Following endotracheal intubation, an ear, nose, and throat (ENT) specialist placed the Boyle-Davis mouth gag. The extension of mouth opening and head position of the patients were adjusted by the same ENT specialist to enhance the exposure of adenoid and tonsillar tissue. When the placement of the mouth gag was completed and the head was positioned for surgery, the operating room (OR) anesthesiologist took a photograph of the neck extension in the lateral view.

**Fig. 1:**
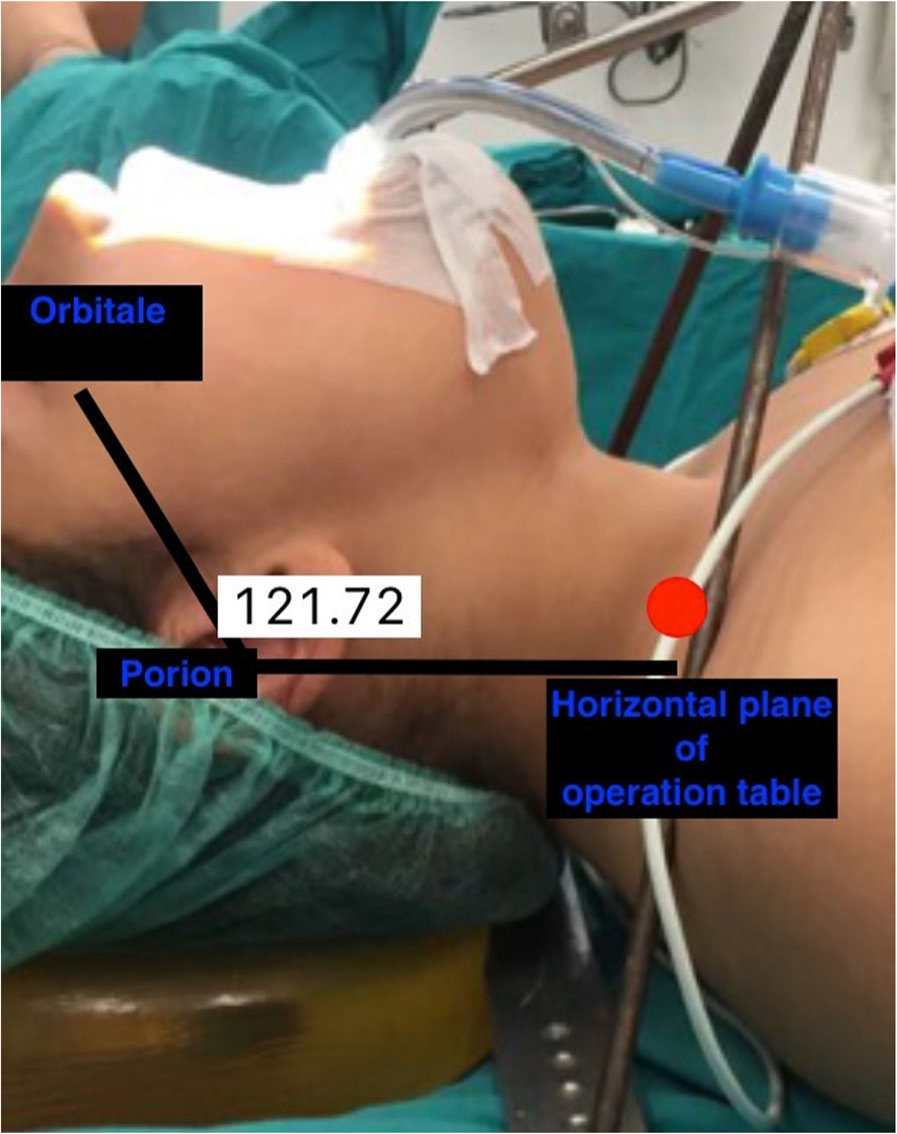
Calculation of the degree of neck extension by measuring the angle between the Frankfort plane (the imaginary line passing from left orbital to left porion point) and horizontal plane of the operation table in the natural position. The degree neck extension is measured 121.72 degrees in this figure

The degree of neck extension was assessed by the angle between the Frankfort plane and horizontal plane of the operation table in the natural position (Frankfort plane angle). The angle was calculated by using a dedicated application (Angles in Photos, 2015 kublaidos) (Fig. 1). The anesthesiologist recorded the average of Frankfort plane angle measurements. A Frankfort plane which was officially described in the anthropologic conference in Frankfort in 1884, is an imaginary line passing from the left orbital to the left porion point. It has been used as a reference plane for cephalometric studies. Recently, the Frankfort plane angle was used for the assessment of neck flexion-extension in the study of Kobayashi et al. [12]. The horizontal plane was created by drawing an imaginary line touching the porion and passing parallel to the operation table while the table was in the neutral position. A very recent study used a similar method to calculate the degree of neck extension to evaluate its effect on ONSD of children undergoing palatoplasty surgery [13].

**Fig. 2:**
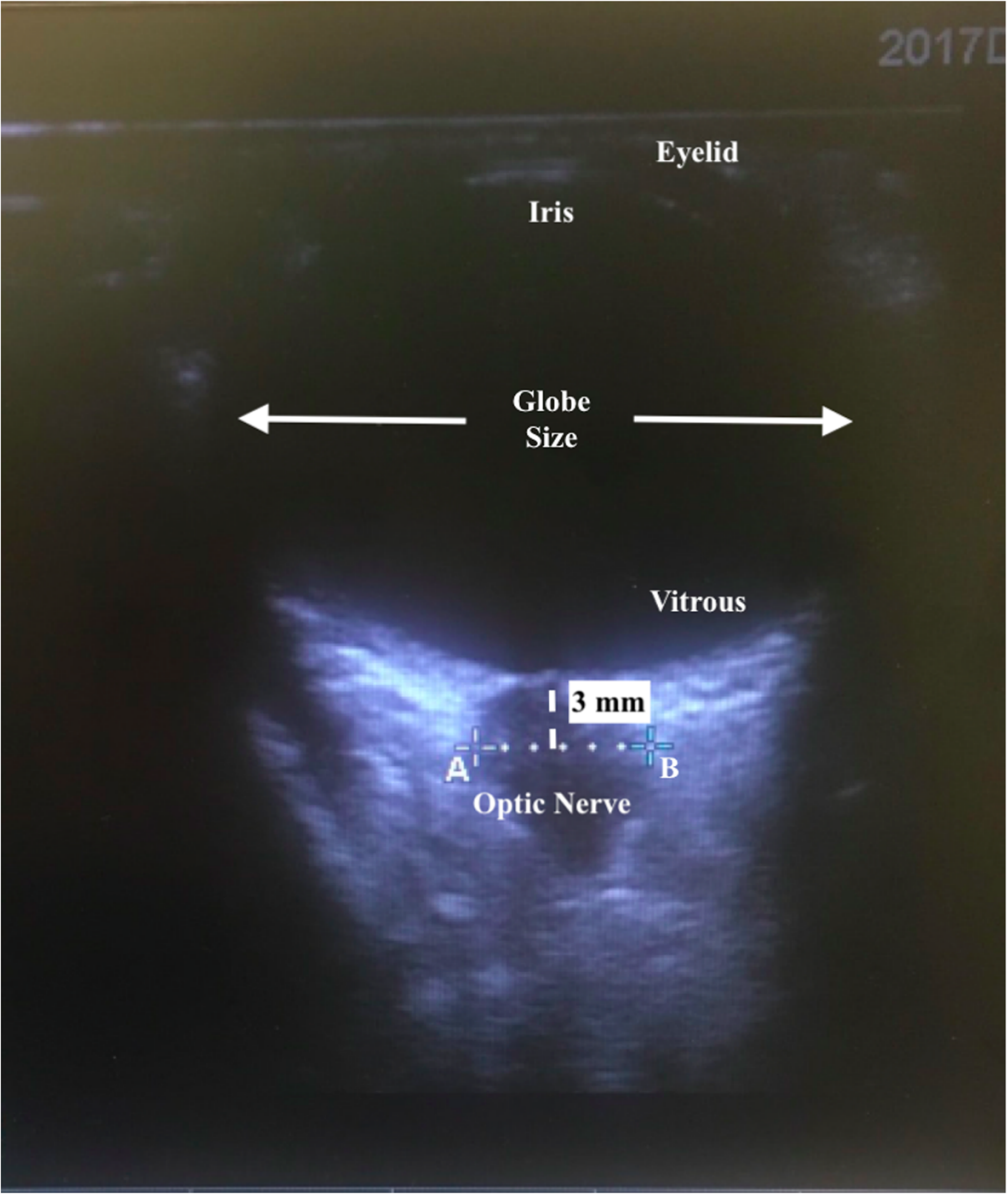
The ultrasonographic view of the axial axis of optic nerve. Optic nerve sheath diameter was measured between the A and B points at 3 mm posterior to the globe

ONSD was measured by two investigators who had experience in over 50 cases. A linear 6–13–Hz probe (Fujifilm Sonosite, Bothwell, USA) was used for the sonographic measurements at four different time-points. A thick layer of water-soluble ultrasound-transmission jelly was applied over the left upper eyelid of each patient. Then, the probe was gently placed over the eyelid without exerting excessive pressure. The probe was moved with careful attention to find the best image of the optic nerve entering into the globe. The ONSD was measured 3 mm posterior to the globe. In addition, the maximum eyeball transverse diameter was also recorded on this plane (Fig. 2). The investigators measured ONSD three times from the same eye and recorded the average of these measurements at four different time points: (T0) after induction of anesthesia, (T1) after endotracheal intubation, (T2) after mouth gag placement, and (T3) 20 min after mouth gag placement. At each time point, heart rate (HR), mean arterial pressure (MAP), EtCO2, and nasopharyngeal temperature (temp) were also recorded. The hemodynamic parameters, temperature, and EtCO2, which are known to be associated with intracranial pressure [14], were maintained in normal ranges in order to minimize their effects on ONSD.

The primary outcome of the study was the change in ONSD measurements between T3 and T4, and the secondary outcome was the effect of the degree of neck extension on mean ONSD changes between T2 and T3 time points.

### Sample size

Power estimation analysis conducted a priori concluded a sample size of 30 with 80% power with alpha error of 0.05; we decided to include 35 patients with the assumption of possible drop-outs. The mean ONSD measurement in the healthy pediatric population is 3.08 ± 0.36 mm. An increase ≥0.3 mm in mean ONSD measurement (10% of the mean ONSD value in healthy pediatric population) was considered clinically significant [15]. Considering a 0.05 significance level for type 1 errors and 0.20 significance level for type two errors, the collected data were sufficient for the power of the statistical tests that were used.

### Statistical analysis

Statistical analyses were conducted using the SPSS version 25 (SPSS Inc., Chicago, Illinois, USA). All continuous variables including age, weight, globe size, ONSD, EtCO_2_, temperature, HR, and MAP are presented as mean ± standard deviation, and the categorical variables, sex, and surgical type, are presented as both numbers and percentile (%). The relationship between the Frankfort plane angle and ONSD changes were analyzed using a regression model. A linear mixed model was used to observe the variation of repeated ONSD measurements and the other parameters (EtCO_2_, temperature, HR, MAP) over time. Post hoc analyses were performed using Bonferroni correction for multiple comparisons, and as pairwise comparisons for time-level because the time wise differences were statistically significant in all parameters observed. Additionally, a figure for parameter changes by time was plotted and presented. A *p* value of < 0.05 was considered significant for the analyses.

## Results

**Fig. 3:**
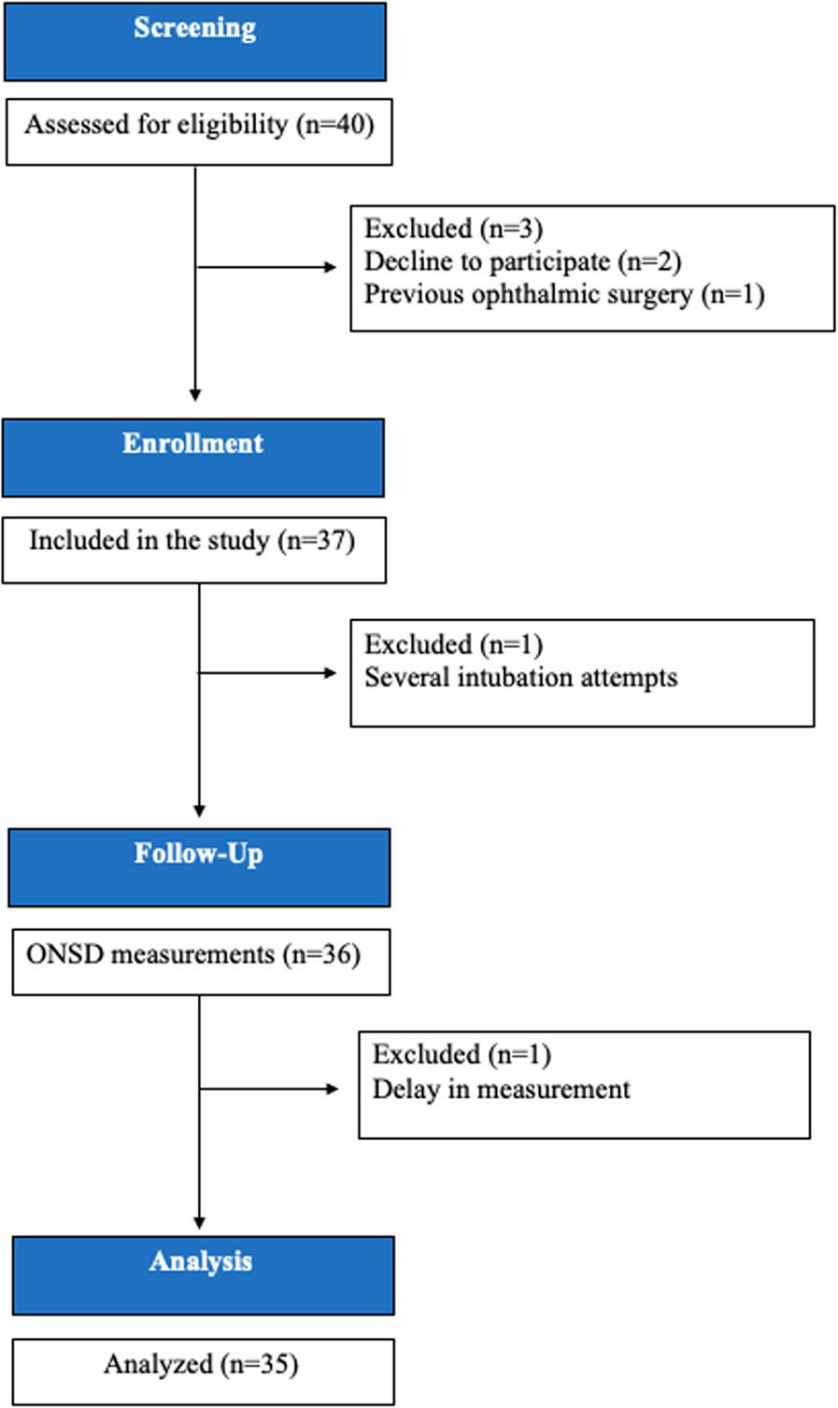
Flowchart of the study

A total of 35 children were included in the study. A description of enrollment is summarized in Fig. 3; 22 were male and the mean age was 7.3 ± 2.75 years. The demographic data of the patients are listed in Table 1.

**Table 1:** Demographic and procedure variables for children undergoing the Boyle-Crowe mouth gag placement

	Tonsillectomy	Adenotonsillectomy
Male / Female (%)	10 (76.9) / 3 (23.1)	12 (54.5) / 10 (76.9)
Age (years)	7.15 ± 2.73	7.41 ± 2.82
Weight (kg)	24.77 ± 9.58	24.86 ± 9.87
Globe Size (mm)	2.16 ± 0.08	2.17 ± 0.14
Number of procedure (%)	13 (37.1)	22 (62.9)

Continues numbers presented as mean ± standard deviation, categorical numbers presented as frequency (percentage)

*T* Tonsillectomy, *AT* Adenotonsillectomy, *SD* standard deviation

The mean ONSD measurement was 4.56 ± 0.41 mm at T0, 5.25 ± 0.58 mm at T1, 5.92 ± 0.63 mm at T2, and 6.28 ± 0.55 mm at T3. The maximum increase in ONSD was after intubation (0.69 ± 0.06 mm) and immediately after mouth gag placement (0.67 ± 0.07 mm). According to the pairwise comparisons (time to time), the mean difference between T0 and T1 was calculated as − 0.07 (CI: − 0.09,-0.05), the difference between T1 and T2 was calculated as − 0.07 (CI: − 0.09,-0.05), and the difference between T2 and T3 was calculated as − 0.04 (CI: − 0.05,-0.02). There were significant differences in all comparisons (*p* < 0.001). The comparisons of mean ONSD values between different time points are listed in Table 2.

**Table 2:** Changes in optic nerve sheath diameter measurements between time points

			95% CI		*p*
Mean ONSD value (mm)	Time	Mean Difference	Lower	Upper	
T0 4.56 ± 0.41	T1	−0.07	−0.09	− 0.05	< 0.001
	T2	−0.14	−0.16	− 0.11	< 0.001
	T3	−0.17	−0.19	− 0.15	< 0.001
T1 5.25 ± 0.58	T0	0.07	0.05	0.09	< 0.001
	T2	−0.07	−0.09	− 0.05	< 0.001
	T3	−0.10	−0.12	− 0.09	< 0.001
T2 5.92 ± 0.63	T0	0.14	0.11	0.16	< 0.001
	T1	0.07	0.05	0.09	< 0.001
	T3	−0.04	−0.05	− 0.02	< 0.001
T3 6.28 ± 0.55	T0	0.17	0.15	0.19	< 0.001
	T1	0.10	0.09	0.12	< 0.001
	T2	0.04	0.02	0.05	< 0.001

Continues numbers presented as mean ± standard deviation

*ONSD***:** Optic nerve sheath diameter, *T0*: After induction of general anesthesia, *T1*: After endotracheal intubation, *T2*: After the mouth gag placement, *T3*: 20 min after the mouth gag placement

*p value was adjusted for multiple comparisons with Bonferroni test, CI: confidence interval

The mean Frankfort plane angle was calculated as 130.41 ± 7.5° (minimum 114.23° and maximum 144.65°). We assessed the relation between the Frankfort plane angle and mean ONSD changes between T2 and T3 time points to evaluate the effect of neck extension on ONSD measurements. According to the regression model, there was no relation between the Frankfort plane angle and mean ONSD changes (β = 0.63, *p* = 0.715).

We summarized the hemodynamic parameters and other variables potentially affecting ICP, and consequently ONSD measurements, in Table 3. According to the Greenhouse-Geisser analysis, EtCO_2_, temperature, HR, and MAP values changed over time. The maximum mean HR (110 ± 17 beats/min) and MAP values (91.2 ± 11.3 mmHg) were recorded immediately after mouth gag placement (T2). Although HR and MAP significantly decreased 20 min after mouth gag use, the mean ONSD measurement increased by 0.36 ± 0.04 mm between T3 and T4 time-points (*p* < 0.001).

**Table 3:** Hemodynamic parameters and other variables associated with intracranial pressure at different time points

Time-point	EtCO_2_ (kPa)	Temp (C°)	HR (beats min^−1^)	MAP (mm Hg)
T0	35.1 ± 2.6	36.5 ± 0.12	99 ± 18	79.6 ± 9.3
T1	36.5 ± 2.6	36.5 ± 0.13	109 ± 16	87.89 ± 10.9
T2	36.9 ± 2.6	36.5 ± 0.11	110 ± 17	91.2 ± 11.3
T3	36.8 ± 2.7	36.5 ± 0.09	97 ± 19	80.2 ± 10.2
Within-level p value	< 0.001	0.005	< 0.001	< 0.001

Continues numbers presented as mean ± standard deviation

*EtCO*_*2*_ end-tidal CO_2_, *Temp* Body temperature, *HR* Heart rate, *MAP* Mean arterial pressure, *T0* After induction of general anesthesia, *T1* After endotracheal intubation, *T2* After the mouth gag placement, *T3* 20 min after the mouth gag placement

*p* value obtained with Greenhouse-Geisser, within-level p value represents the time wise change in overall

## Discussion

In the current study, we evaluated the effects of mouth gag placement and the degree of neck extension on ONSD measurements. We detected significant increases in ONSD immediately after mouth gag placement, and additionally, ONSD values continued to rise 20 min after mouth gag use. However, the degree of neck extension, as assessed using the Frankfort plane angle, had no effect on ONSD measurements.

Previously, Padayachy et al. [16] analyzed 174 children aged over 1 year. The authors aimed to calculate the optimal cut-off value of ultrasonographic measurement of ONSD for detecting an increased ICP. They calculated that ONSD values > 5.49 mm were an indicator of ICP ≥15 mmHg with a sensitivity of 93.7%, specificity of 74.4%, and ONSD values > 5.75 mm were an indicator of ICP ≥20 mmHg with a sensitivity of 85.9%, and specificity of 70.4%. In our study, the mean ONSD value was measured as 5.92 ± 0.63 mm immediately after mouth gag placement, and ultimately, it increased to over 6.0 mm (6.28 ± 0.55 mm) only 20 min after mouth gag placement. According to data provided in previous studies [8, 16], the children in our study probably had elevated ICP 20 min following the mouth gag use. An et al. [3] showed that mouth gag placement for exposure of pharyngeal tonsils during T and AT surgeries caused significant increases in HR and MAP measurements of pediatric patients. The authors stated that these hemodynamic changes were similar to the hemodynamic response caused by direct laryngoscopy. According to these data, we hypothesized that mouth gag placement during surgery would cause a significant increase in ONSD measurements, and ONSD values would probably remain increased as long as the mouth gag use continued. In our study, the mean ONSD value increased by 0.36 ± 0.04 mm and passed the pathologic cut-off values 20 min after mouth gag placement.

The hemodynamic responses caused by laryngoscopy are believed to be induced by the direct contact of the blade with the posterior third of the tongue, manipulation of the richly innervated epiglottis, and insertion of the endotracheal tube between the vocal cords [17]. During mouth gag placement, a similar blade is in direct contact with the tongue and the suspension system causes contractions in the muscles of mastication. As the oropharynx is a sensory organ capable of initiating sympathetic reflexes [18], the catecholamine release due to mouth gag use is not surprising. However, we found the effect of mouth gag placement on ONSD measurements much more significant than the effect of direct laryngoscopy. Furthermore, mean ONSD values continued to rise as long as the gag remained in the mouth. Increased ICP is known to reduce cerebral perfusion pressure and regional oxygenation, which may result in postoperative neurologic complications [19]. Although the mouth gag remains for a short time in otherwise healthy children during A and AT surgeries, it has to be kept in the mouth for significantly longer durations during other procedures such as cleft palate repair, obstructive sleep apnea surgery, and intraoral tumor excision. Consequently, long-term placement of the mouth gag may cause deleterious results, especially in patients with comorbidities during longer procedures. A time limitation might be concerned for the duration of the mouth gag use.

Previously, Panjabi et al. [20] reported that a rotation of the upper cervical spine over 20° in the sagittal plane exceeded the normal range of physiologic motion. Erden et al. [21] reported that endotracheal intubation with a Macintosh blade caused a maximum of 19.4° movement in the C1/C2 spine. Thus, excessive extension of the neck during mouth gag use is likely to exceed the normal ranges of cervical motion. We evaluated the effect of neck extension angle on ONSD measurements. However, we detected no relation between the degree of neck extension and ONSD measurements of children according to the regression model.

Some intraoperative factors such as hemodynamic parameters, EtCO_2_ and nasopharyngeal temperature may affect ONSD by changing ICP. An increase in carbon dioxide causes an increase in ICR by dilating the blood vessels, whereas decreases in carbon dioxide or the presence of hypothermia cause a reduction in ICP and probably ONSD [14, 22]. In the current study, although time-wise differences were detected in EtCO_2_, temperature, and hemodynamic parameters, each was within the normal range throughout the surgeries. Intraoperative parameters were not thought to be the main reason for increased ONSD values.

The main limitation of the study is that we could not evaluate the postoperative impacts of increased ONSD. All participants were healthy children and the duration of the mouth gag use was relatively short compared with other types of surgery. Procedures with longer duration of mouth gag placement such as intraoral tumor excision would be more helpful in determining postoperative cognitive deteriorations. However, postoperative cognitive deterioration was not an outcome of the current study. Secondly, we could not measure the ONSD of the children prior to induction. The mean age of the children was 7 years; therefore, we could not assess ONSD while the patients were awake. As a result, we could not provide pre-operative data. In order to compensate for this limitation, we defined the primary outcome as changes in ONSD measurements between the mouth gag placement and 20 min after mouth gag placement. We did not consider the changes between the first (following induction of anesthesia) and the last (20 min after mouth gag placement) measurements. Lastly, the assessment of neck extension was based on the Frankfort plane angle, which was calculated using a dedicated phone application. The lines passing through the Frankfort plane and horizontal plane of the operation table in the natural position were drawn manually. Although we measured the angle three times for each patient, miscalculation was still possible.

## Conclusion

Placement of a mouth gag causes significant increases in the ONSD measurements of children. Therefore, attention to the duration of mouth gag placement should be considered during surgery.

## Data Availability

Additional data available from the corresponding author on reasonable request.

## Abbreviations

ONSD: Optic nerve sheath diameter
T: Tonsillectomy
AT: Adenotonsillectomy
ICP: Intracranial pressure
IOP: Intraorbital pressures
ASA: American Society of Anesthesiologists
BIS: Bi-spectral index
EtCO_2_: End-tidal CO_2_
ENT: Ear-nose-throat
OR: Operating room
HR: Heart rate
MAP: Mean arterial pressure
SPSS: Statistical Package for the Social Sciences
Temp: Nasopharyngeal temperature
